# Temporal and spatial analysis of event-related potentials in response to color saliency differences among various color vision types

**DOI:** 10.3389/fnhum.2024.1441380

**Published:** 2024-10-02

**Authors:** Naoko Takahashi, Masataka Sawayama, Xu Chen, Yuki Motomura, Hiroshige Takeichi, Satoru Miyauchi, Chihiro Hiramatsu

**Affiliations:** ^1^Graduate School of Design, Kyushu University, Fukuoka, Japan; ^2^Graduate School of Information Science and Technology, The University of Tokyo, Tokyo, Japan; ^3^Faculty of Design, Kyushu University, Fukuoka, Japan; ^4^Open Systems Information Science Team, Advanced Data Science Project, RIKEN Information R&D and Strategy Headquarters, RIKEN, Kanagawa, Japan; ^5^Department of Physiology, Kansai Medical University, Osaka, Japan

**Keywords:** color vision, diversity, anomalous trichromacy, saliency, EEG, ERPs

## Abstract

**Introduction:**

Human color vision exhibits significant diversity that cannot be fully explained by categorical classifications. Understanding how individuals with different color vision phenotypes perceive, recognize, and react to the same physical stimuli provides valuable insights into sensory characteristics. This study aimed to identify behavioral and neural differences between different color visions, primarily classified as typical trichromats and anomalous trichromats, in response to two chromatic stimuli, blue-green and red, during an attention-demanding oddball task.

**Methods:**

We analyzed the P3 component of event-related potentials (ERPs), associated with attention, and conducted a broad spatiotemporal exploration of neural differences. Behavioral responses were also analyzed to complement neural data. Participants included typical trichromats (*n* = 13) and anomalous trichromats (*n* = 5), and the chromatic stimuli were presented in an oddball paradigm.

**Results:**

Typical trichromats exhibited faster potentiation from the occipital to parietal regions in response to the more salient red stimulus, particularly in the area overlapping with the P3 component. In contrast, anomalous trichromats revealed faster potentiation to the expected more salient blue-green stimulus in the occipital to parietal regions, with no other significant neural differences between stimuli. Comparisons between the color vision types showed no significant overall neural differences.

**Discussion:**

The large variability in red-green sensitivity among anomalous trichromats, along with neural variability not fully explained by this sensitivity, likely contributed to the absence of clear neural distinctions based on color saliency. While reaction times were influenced by red-green sensitivity, neural signals showed ambiguity regarding saliency differences. These findings suggest that factors beyond red-green sensitivity influenced neural activity related to color perception and cognition in minority color vision phenotypes. Further research with larger sample sizes is needed to more comprehensively explore these neural dynamics and their broader implications.

## 1 Introduction

Color perception is a subjective experience unique to each individual. Colors serve diverse functions, including aiding in object detection and recognition for prompt environmental understanding (Conway et al., 2023) and enriching the qualitative aspects of perception through aesthetics or symbolic meanings (Elliot and Maier, 2007; Muratbekova and Shamoi, 2024). Despite their internal nature, colors are communicated through color names in everyday discourse (Berlin and Kay, 1991; Kay and Regier, 2003). However, sharing subjective experiences can be more abstract, particularly when individuals with diverse sensory characteristics are involved (Hiramatsu et al., 2023). Therefore, characterizing the neural mechanisms underlying diverse color experiences across various types of color vision remains an important challenge in the visual neuroscience.

Variations in color vision are a common genetic trait in human population, with a small percentage of individuals (predominantly males due to the X-chromosome-linked inheritance) exhibiting minority color vision phenotypes that differ from the prevalent type of trichromacy, hereafter referred to as “typical trichromacy,” based on S, M, and L cone photoreceptor cells (Birch, 2012; Neitz and Neitz, 2011; Deeb, 2005; Asenjo et al., 1994; Nathans et al., 1986). Most of these variations result from altered photosensitivity, particularly in the L and M cone cells. Approximately 6% of Caucasian males (Birch, 2012) and 3% of Asian males (Okajima, 2011) have anomalous trichromacy due to shifts in photosensitivity in one class of cone cells, while ~2% have dichromatic color vision based on two classes of cone cells.

Variations in L and M cone sensitivities result in confined red-green color discrimination. The responses of the L and M cone cells are compared early in visual processing, and the relative outputs of these cone contrast form the basis of the cardinal red-green color axis (Werner and Wooten, 1979). Generally, sensitivity shifts reduce perceived chromatic differences due to diminished contrast gains from the L/M comparison (Pokorny and Smith, 1977). In anomalous trichromacy, the spectral sensitivity separation between L and M cones ranges between 1 and 12 nm (Merbs and Nathans, 1992; Asenjo et al., 1994; Neitz and Neitz, 2011), whereas in typical trichromacy, the separation is ~25 nm (Dartnall et al., 1983; Merbs and Nathans, 1992; Stockman and Sharpe, 2000). In dichromacy, the absence of the L/M contrast results in the absence of the red-green color axis, and color vision is theoretically based on the blue-yellow axis, which derived from comparing S cone responses against L and M cone signals. However, the actual color-discrimination capacities of individuals with anomalous trichromacy and dichromacy do not always align with the anticipated color perception based on their specific cone sensitivities (Bosten, 2019). A higher red-green sensitivity than expected based on cone sensitivity has been reported in a wide range of studies, including psychophysical color-matching experiments and color-naming tasks with cognitive components (Scheibner and Boynton, 1968; Smith and Pokorny, 1977; Nagy and Boynton, 1979; Montag, 1994; Neitz et al., 1999). For example, in a color-naming experiment, dichromats exhibited color categorization capabilities similar to typical trichromats when stimuli were presented with sufficient size and time for identification (Montag, 1994).

Several mechanisms have been suggested to explain the extended red-green sensitivity, such as rod contributions providing alternative signals (Smith and Pokorny, 1977; Nagy and Boynton, 1979; Montag and Boynton, 1987), enhancement of S-cone sensitivity in long-wavelength regions serving as alternative signals (Scheibner and Boynton, 1968), and variation in optical density (Neitz et al., 1999; Thomas et al., 2011). A recent psychophysical study suggested post-receptoral enhancement (Boehm et al., 2021), and physiological studies have reported binocular facilitation of visual evoked potentials (Rabin et al., 2018) and enhanced neural activity in the early visual cortex (V2; Tregillus et al., 2021) in anomalous trichromacy, even during passive viewing. Overall, current evidence suggests that multiple mechanisms contribute to the extended sensitivity observed in anomalous trichromacy and dichromacy.

Despite the significant perceptual diversity among trichromats (Barbur and Rodriguez-Carmona, 2017; Barbur et al., 2021), few studies explore how this diversity is reflected in cognitive processes in the brain. Therefore, we aimed to characterize the patterns of spatiotemporal neural activity in different color vision types during a sequence of neural activities involved in perceptual-cognitive processing. Specifically, we investigated neural activity measured by electroencephalography (EEG) during an attention-demanding oddball task, as attention is an indispensable cognitive mechanism used in everyday visual tasks where chromatic differences often serve as cues for distinguishing objects from their background.

In the oddball task, we asked participants with different red-green sensitivities to detect rarely presented deviant stimuli, blue-green and red, from a frequently presented standard stimulus, green. The stimulus chromaticities were selected so that the predicted saliencies of the deviant stimuli were reversed between color vision types—red was more salient for typical trichromats, while blue-green was more salient for dichromats and anomalous trichromats with large red-green sensitivity thresholds. Using chromatically identical stimuli for all participants allowed us to directly compare the effects of stimulus conditions and differences in chromatic sensitivity.

The P3 component of event-related potentials (ERPs), which reflects allocated attentional resources (Kramer et al., 1986; Näätänen, 1988; Gray et al., 2004), was analyzed as a primary indicator of attention-related neural activity during stimulus processing. We hypothesized that P3 amplitude would be higher for the less salient stimulus due to increased cognitive resource allocation and that P3 latency would be shorter for the more salient stimulus, reflecting faster processing due to the higher saliency. The same trend was also expected from reaction times (RTs).

The effects of chromatic sensitivity and stimulus condition on P3 amplitude and RTs were examined while controlling for random factors. Additionally, spatiotemporal differences in neural responses between stimulus conditions and color vision types were explored to identify any facilitation that was specific to each color vision type.

In summary, this study seeks to provide a broader understanding of how variations in chromatic sensitivity influence neural and behavioral responses during attention-demanding tasks. By examining these effects, we aim to contribute insights into the neural mechanisms underlying diverse color perception and cognitive processing across different color vision phenotypes.

## 2 Materials and methods

### 2.1 Participants

Nineteen male participants with normal or corrected-to-normal visual acuity participated in this study, most of whom were undergraduate and graduate students at Kyushu University School of Design (mean age ± standard deviation: 23.32 ± 2.68 years). Only males were included in the study because ~10% of females carry variant red and green visual pigment genes, which can modify cone sensitivity, with uncertain effects on color perception (Jordan et al., 2010).

To determine each participant's color vision type, four color vision tests were conducted: the Ishihara pseudoisochromatic plate test, Color Assessment and Diagnosis (CAD) test (Barbur et al., 2021), the HMC-anomaloscope (Oculus), and the Farnsworth—Munsell 100 hue test. Based on the combined results, five participants were identified as deuteranomalous trichromats (anomalous trichromacy with altered sensitivity in the M cone), and one as a deuteranope (dichromacy without the M cone), while the remaining were typical trichromats.

Participants received financial compensation for their participation. The experimental procedure adhered to the Declaration of Helsinki, and was approved by the Ethics Committee of the Graduate School of Design at Kyushu University (Approval No. 316). Written informed consent was obtained from each participant prior to the experiment.

To account for individual variability in color perception, red-green color sensitivity was measured using the CAD test. Table 1 shows measured red-green thresholds of deuteranomalous and deuteranope participants. These thresholds represent the amount of saturation required to perceive the color from the neutral gray point along the evaluation color axis, where a threshold of 1 represents the average discrimination threshold of typical trichromats (Barbur and Rodriguez-Carmona, 2017; Barbur et al., 2021).

**Table 1:** Red-green thresholds assessed using the CAD test and identified color vision types based on the anomaloscope test for participants with minority color vision phenotypes.

**Participant code**	**Red-green threshold**	**Color vision type**
Participant 14	21.63	Deuteranomalous trichromacy
Participant 07	19.36	Deuteranopia
Participant 02	19.35	Deuteranomalous trichromacy
Participant 18	13.34	Deuteranomalous trichromacy
Participant 03	9.07	Deuteranomalous trichromacy
Participant 16	2.19	Deuteranomalous trichromacy

Participants are listed in descending order according to their red-green threshold.

In this study, the average threshold for typical trichromats was 1.1 ± 0.2, while the average threshold for anomalous trichromats was 13.1 ± 7.9.

### 2.2 Stimuli

**Figure 1:**
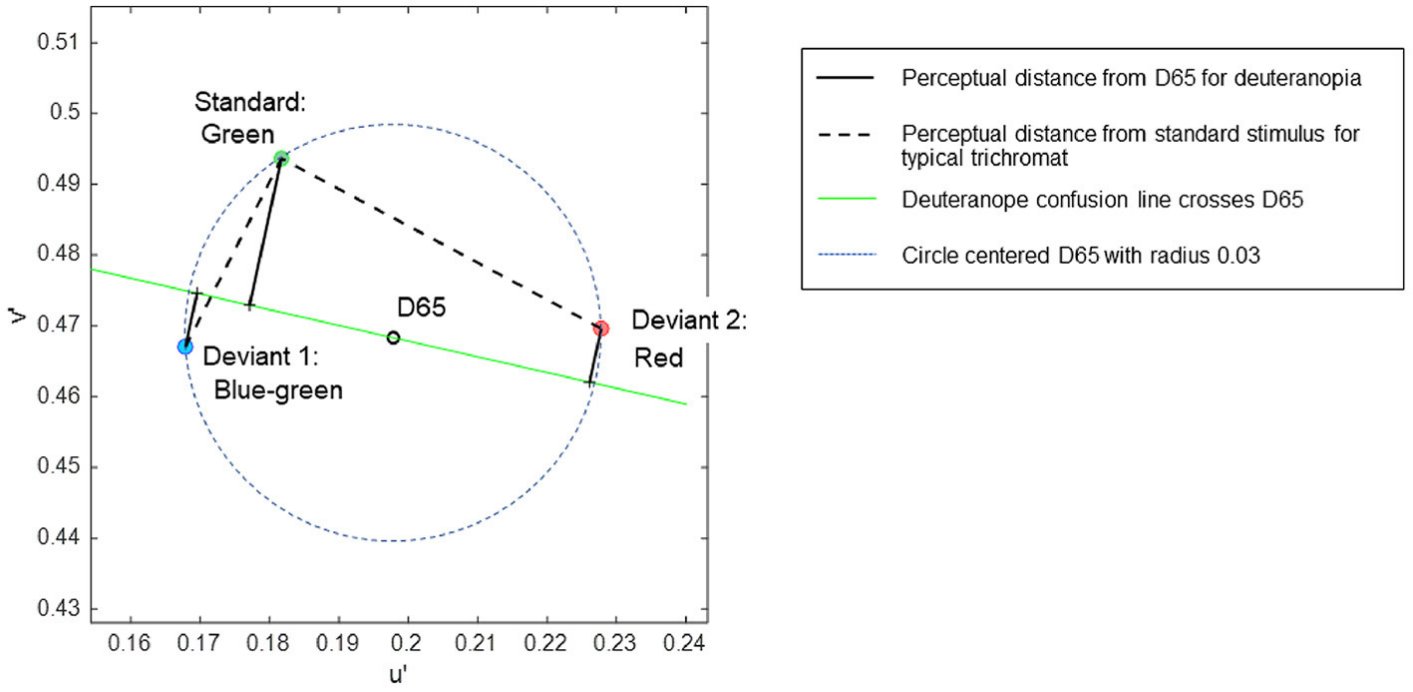
The coordinates of stimuli in the CIE 1976 u', v' uniform chromaticity scale diagram, in which perceptual distance is represented as Euclidean distance for typical trichromats. The expected chromatic contrasts between the standard and deviant stimuli are represented as line lengths: black dotted lines represent typical trichromacy, and black solid lines represent deuteranopia.

A set of color stimuli was used to convey different chromatic contrasts when each was paired with a common standard stimulus. The chromaticities of the stimuli were selected based on the CIE 1976 u', v' uniform chromaticity scale diagram, allowing for the estimation of perceptual chromatic distances among the stimuli (Pointer, 1981). Three colors—blue-green, orange-tinted red (referred to simply as red), and green—were selected to be equidistant from the neutral gray of D65 in the u', v' diagram (Figure 1). The specific coordinates of the stimuli were (0.1679, 0.4670) for blue-green, (0.2278, 0.4696) for red, and (0.1817, 0.4936) for green, each situated at an equal distance (0.03) from D65 (0.1978, 0.4683), ensuring that each stimulus had equal saturation relative to D65. In the attention task, the target (deviant) stimuli were blue-green or red, in contrast to the standard green stimulus.

In the uniform color space, perceptual differences in colors are represented as Euclidean distances for typical trichromacy; the greater the distance between two points, the larger the difference in perception. The Euclidean distances of blue-green and red from green in the space were 0.03 and 0.052, respectively. For typical trichromacy, the red pair was expected to be more salient than the blue-green pair because the Euclidean distance from standard green was greater for red than for blue-green. This relationship can also be explained by categorical color differences: blue-green and green are expected to be categorically more similar than red and green in typical trichromacy. Thus, visual saliency was expected to be greater for red than for blue-green when contrasted with green, and categorical facilitation might influence performance (Witzel and Gegenfurtner, 2015).

The color space of minority color vision phenotypes is still debated (Broackes, 2010). However, theoretical estimates of relative chromatic contrasts between deviant and standard stimuli for deuteranopia can be obtained. In deuteranopia, colors that align approximately along the red-green axis in typical trichromacy appear to be the same, forming confusion lines on a chromaticity diagram. According to Pridmore (2014), the empirical color axis in dichromats is orthogonal to the confusion line, intersecting the neutral gray point. As such, the chromatic distances between colors for deuteranopia can be represented as the distance along a line orthogonal to the confusion line passing through the neutral gray point D65 (Figure 1).

Additionally, the confusion line intersecting D65 is considered as the categorical boundary for dichromacy (Broackes, 2010), where points on the same side of the boundary denote categorical similarity. Hence, the chromatic difference can be calculated as the sum of the orthogonal distances to the confusion line when the stimuli are positioned on opposite sides of the line. Conversely, when stimuli are on the same side, the chromatic difference is calculated by subtracting these distances.

The relative chromatic contrasts of the blue-green and red deviants to the green standard stimulus for deuteranopia were estimated to be 0.0290 and 0.0134, respectively. These estimations were based on the orthogonal distances of 0.0212, 0.0078, and 0.0078 for green, blue-green, and red, respectively, with the confusion line depicted in Figure 1. Therefore, blue-green was expected to be more salient than red when contrasted with green for individuals with deuteranopia and anomalous (deuteranomalous) trichromacy close to deuteranopia. However, this was not guaranteed for all anomalous trichromats, particularly for participants with a low red-green threshold (Table 1). Categorically, red and green were expected to be more similar than blue-green and green in deuteranopia and anomalous trichromacy, with a high red-green threshold.

These theoretical estimates further predict a reversal in the saliency of deviant stimuli in typical trichromacy and deuteranopia, as the ratio of blue-green to red to the distance from green was 0.03/0.052 = 0.57 for typical trichromacy, whereas it was 0.029/0.0134 = 2.16 for deuteranopia, although the absolute perceived contrasts were difficult to estimate.

Given the diversity of red-green thresholds across participants with anomalous trichromacy, chromatic contrasts between stimuli would naturally differ among participants. However, we prioritized the use of the same chromatic stimuli for all participants, rather than adjusting the chromatic contrast across participants. This approach allowed us to observe the neural diversity associated with differences in the perception of stimuli of the same color. To ensure that luminance contrast was not used as a cue for the task, the luminance of the color stimuli was equated to 20 *cd*/*m*^2^ D65 for each participant using flicker photometry (see 2.5 Procedure for details).

### 2.3 Task design

**Figure 2:**
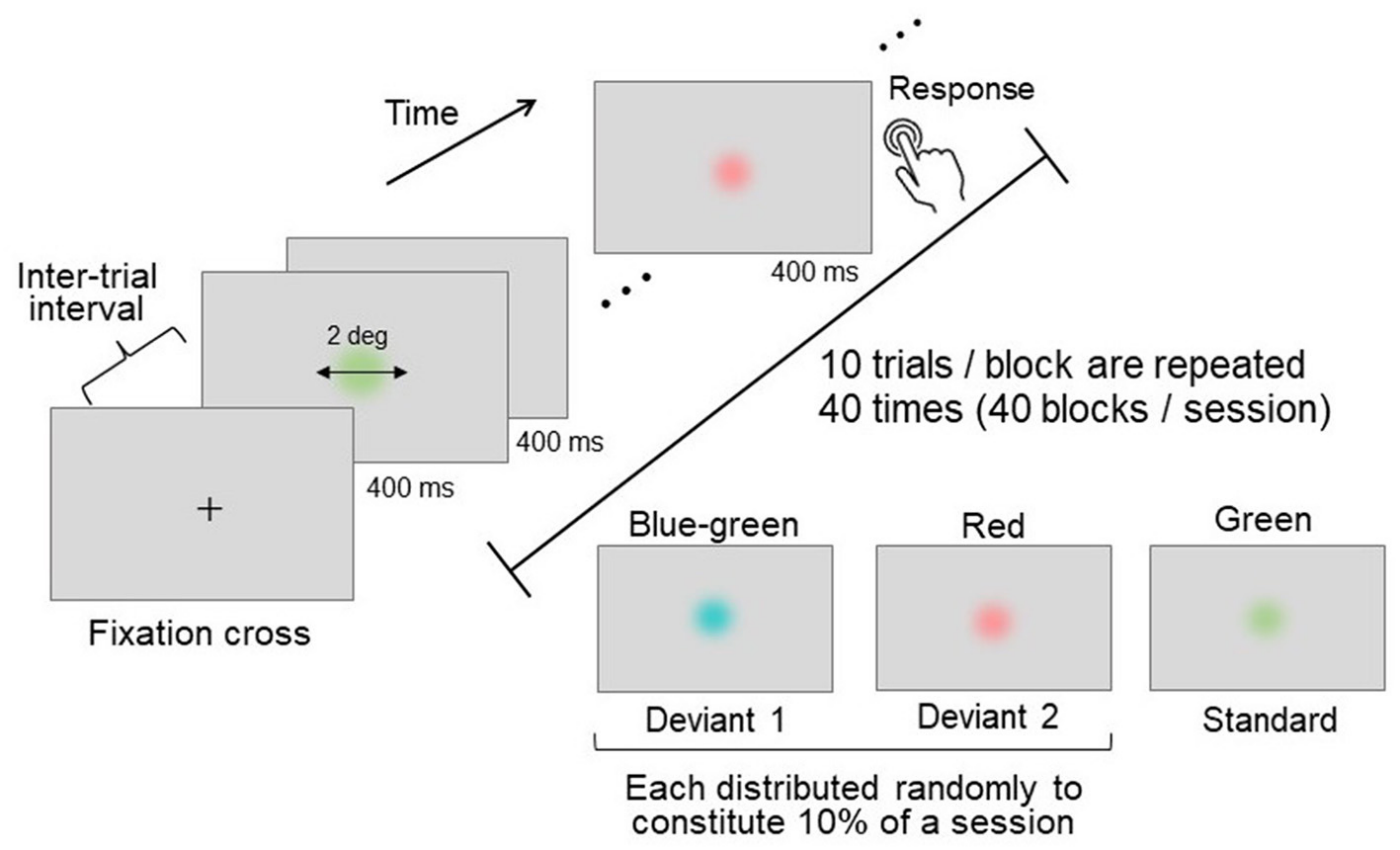
Schematics of the oddball task. Three distinct colors were used: two deviant stimuli (presented rarely) and one standard stimulus (presented frequently). Each stimulus was presented for a duration of 400 ms, with an inter-trial interval of 1,200–1,600 ms. Participants were instructed to press a button immediately upon detecting a deviant stimulus.

An oddball paradigm task (Sutton et al., 1965; Picton, 1992) was employed as the attention-demanding task. In this task, a frequently appearing standard stimulus and a rarely appearing deviant stimulus were presented in a random sequence. The participants were asked to detect the deviant stimulus (either blue-green or red) among the standard stimuli (green) and to respond by pressing a button immediately upon detection (Figure 2).

The selection of blue-green as deviant 1 and red as deviant 2 was based on the estimated chromatic contrasts relative to the standard stimulus, aiming to evoke varying levels of attention. Furthermore, the chromatic contrast relationship between the standard and deviant pairs was designed to be reversed between typical trichromacy and deuteranopia (see 2.2 Stimuli).

### 2.4 Apparatus

The stimuli were displayed on a linearity-calibrated LCD monitor (Display++, Cambridge Research Systems Ltd.), paired with an image processor (Bits#, Cambridge Research Systems Ltd.), capable of presenting colors at 14-bit resolution with a 120 Hz refresh rate. The chromaticity and luminance of the stimuli were verified using a spectroradiometer (SR-LEDW-5N, Topcon Technohouse). The experimental program was controlled using MATLAB (MathWorks Inc.) with the psychtoolbox-3 extension (Brainard and Vision, 1997; Pelli and Vision, 1997; Kleiner et al., 2007).

EEG signals were recorded using a 64-channel digital recorder (ActiCHamp Plus, Brain Products GmbH). Active electrodes were placed on the scalp following the 10–20 layout system layout, using an electrode cap (actiCap slim, Brain Products GmbH). The timing of the stimulus onset and behavioral responses were recorded through an EEG amplifier equipped with a trigger box via a parallel port. The stimulus onset and stimulus color conditions in each trial were detected using photoelectric sensors (MaP1180PS2A, NIHON SANTEKU Co., Ltd) attached directly to small areas at the edge of the display. These sensors captured binary luminance modulations synchronized with the stimulus. Participants submitted their behavioral responses using a button connected to the trigger box.

### 2.5 Procedure

After the electrode cap was placed on their scalp, each participant was seated 57 cm from the display surface with their eyes fixated at the center of the screen in a dark room. Prior to the oddball experiment, the luminance of the three stimulus colors (blue-green, red, and green) was adjusted to match 20 *cd*/*m*^2^ D65, the color used as the background, using flicker photometry (Wagner and Boynton, 1972; Kaiser and Comerford, 1975; Anstis and Cavanagh, 1983) with a 15 Hz alteration. Equiluminance measurements were conducted six times for each stimulus using the up-down method. The starting luminance was either 23 *cd*/*m*^2^ or 17 *cd*/*m*^2^, with the adjustment direction alternating between ascending and descending toward 20 *cd*/*m*^2^ D65 for each measurement. The average luminance values from these measurements were used to set the stimulus luminance levels for subsequent oddball experiments.

During each oddball trial, a disk-shaped stimulus was presented at the center of the screen for 400 ms, with random inter-trial intervals ranging between 1,200 and 1,600 ms (Figure 2). The stimulus had a visual angle of 2°, and its edge was linearly and gradually blended into the gray D65 background to prevent the chromatic edge from influencing stimulus detection.

Each block consisted of ten consecutive trials. The 40 blocks were grouped into a single session, and two sessions were conducted for each participant. The standard-to-deviant stimulus presentation ratio was 8:2, with an equal ratio between the two deviants. The trials featuring deviant stimuli were randomly distributed throughout each session. Although some blocks could contain more than one deviant trial due to randomization, not all blocks necessarily included deviant trials. To avoid confusion, only one type of deviant stimulus was presented per block. In addition, the appearance of a deviant stimulus was always preceded by a standard stimulus, ensuring that the discrimination of deviants was based on the contrast to the standard stimulus. This systematic presentation facilitated the comparison of responses to deviant and standard stimuli.

To ensure clear stimulus instructions and avoid uncertainty, color names were not used to designate stimulus types (standard or deviant). Instead, participants recognized deviant and standard stimuli by observing example stimulus sequences prior to the task. After identifying the stimuli based on their appearance frequency, participants completed three practice blocks to familiarize themselves with the task procedure. Following the practice phase, an experimental phase consisting of two sessions (80 blocks in total) was conducted.

Participants were allowed arbitrary breaks between blocks and could advance to the next block by pressing the space key. A mandatory 10-min break was scheduled between sessions. In total, 800 trials were conducted (640 standard stimulus trials [green], 80 deviant 1 [blue-green], and 80 deviant 2 [red] trials). During the trials, most participants used their right thumb to press the button to record their RTs immediately after detecting deviant stimuli.

### 2.6 Behavioral data analysis

The analysis of RTs was conducted using linear mixed-effects (LME) models to assess the influence of chromatic sensitivity variations, as well as the main effects of stimulus condition and their interactions, while controlling for random variation among participants.

To examine the relative contributions of categorical color vision type and the red-green threshold to RTs, model-based comparisons were performed (Buscemi and Plaia, 2020). LME models were applied to the data from typical trichromats and anomalous trichromats. In these models, fixed effects included color vision type (typical or anomalous trichromats) or the red-green threshold identified by the CAD test, as well as stimulus condition (deviant 1 or deviant 2), with participants included as a random effect to account for individual variability. Trial-based RTs were log-transformed to improve interpretability through normalization (Schielzeth, 2010). Although the red-green threshold is a continuous variable, it exhibited large gaps and differences in distribution scale between color vision types. Therefore, it was z-scored (Lundberg, 2007) to normalize and standardize this variable across color vision types.

A simple model excluding all fixed effects except stimulus condition was initially formulated. This model was compared to models incorporating either color vision type or the red-green threshold as fixed effects.

Simple: Trial-based RTs ~ Condition + (1 ∣ Participants).
Color vision: Trial-based RTs ~ ColorVision * Condition + (1 ∣ Participants).
Red-green threshold: Trial-based RTs ~ RGthreshold *
  Condition + (1 ∣ Participants).

In these models, ColorVision represents color vision type (typical trichromats or anomalous trichromats), RGthreshold represents the red-green threshold, and Condition represents stimulus condition (deviant 1 or deviant 2) as fixed effects. Participants were included as random intercepts (1 ∣ Participants) to account for random effects.

The influence of color vision type and red-green threshold on the simple model was assessed based on likelihood ratio test (Vuong, 1989), and model fit was evaluated with AIC, BIC, and Log-Likelihood values (Aho et al., 2014; Pinheiro and Bates, 1995).

In order to visually assess the individual performances, mean log-transformed RTs were computed based on trial-based records for each stimulus condition. While the deuteranopic individual was excluded from group-based analyzes using LME model due to limited sample size, paired *t*-test was conducted using log-transformed trial-based data.

To evaluate overall behavioral performance, group mean hit rates were computed for deviants 1 and 2, while group mean false alarm rates were computed for the non-target standard stimulus. RTs above 900 ms were identified as outliers and excluded from the behavioral analysis.

All statistical analyzes were conducted using MATLAB software (MathWorks, Inc.).

### 2.7 EEG recording and data analysis

#### 2.7.1 Data preprocessing

EEGs were recorded and digitized at a sampling rate of 1,000 Hz. Subsequent analyzes were conducted using EEGLAB (version 2022.0; Delorme and Makeig, 2004) in MATLAB (MathWorks, Inc.). The data were organized following the Brain Imaging Data Structure (BIDS; Gorgolewski et al., 2016), with the extention of EEG data (Pernet et al., 2019). Initially, the data were bandpass-filtered between 0.5 and 30 Hz. Two EEGLAB plugins, IC Label and Clean Rawdata, were employed to enhance data readability and quality. The IC Label function differentiates independent components (ICs) stemming from brain activity from non-brain sources. ICs related to eye movements, muscle artifacts, or specific channels with >90% probability were marked for rejection. In addition, the Clean Rawdata plugin identified and removed data segments and channels that were significantly contaminated by noise. We adhered to the rejection criteria recommended by Delorme (2023), and supplemented this process by interpolating deleted channels with data from neighboring preserved channels, thereby optimizing the data quality before referring to the average. In the final preprocessing step, 1,000 ms of individual trial data were extracted from the recordings. The average of the 100 ms pre-stimulus period was subtracted to establish the 0 μV baseline (Murray et al., 2008; Luck, 2014; Keil et al., 2014).

#### 2.7.2 Data analysis

The EEG data were segmented into single-trial data with a duration of 1,000 ms post-stimulus onset. These segments were averaged for individual participants and then grand-averaged across participants to compute the ERPs for each stimulus condition within each color vision type (typical and anomalous trichromats). In addition, 95% confidence intervals (CIs) were computed to represent the variation among participants.

To assess stimulus-induced brain activity, the average ERPs at the centroparietal electrodes (Cz, CPz, and Pz) were analyzed with respect to the P3 component. Given the attention-demanding nature of the visual oddball task, we expected that typical attention-related ERP components, such as P3, would be observed at these electrodes (Zhang and Kappenman, 2024), assuming the experimental paradigm was successful. P3 is a late positive component that typically appears ~400 ms post-stimulus onset in response to a rare stimulus within a sequence of stimuli. It tends to be heightened in response to more difficult stimuli (Polich, 1987; Alho et al., 1992), reflecting an increase in the allocation of attentional resources (Grasso et al., 2009; Isreal et al., 1980). In this experimental design, deviant 1 (blue-green) was expected to be more difficult for typical trichromats, while deviant 2 (red) was expected to be more difficult for anomalous trichromats.

The relationship between stimulus condition and P3 amplitude was investigated using LME models. Given that sensitivity diversity is a key characteristic of anomalous trichromats, we assessed its impact on neural representation using likelihood ratio tests. Similar to the approach in the RT analyzes, we formulated a simple model that assumed no influence of chromatic sensitivity diversity on P3 amplitude. This model was then compared against models that accounted for chromatic sensitivity, either by specifying color vision type or by red-green threshold. The models were specified as follows:

Simple: P3 ~ Condition + (1 ∣ Participants).
Color vision: P3 ~ ColorVision * Condition + (1 ∣ Participants).
Red-green threshold: P3 ~ RGthreshold * Condition + (1 ∣ Participants).

In addition to analyzing centroparietal activity, the average ERPs at the frontal (AF3, AFz, and AF4) and occipital (PO7, O1, O2, and PO8) electrodes were visually inspected to confirm the time course at these scalp locations.

Differences in the perceptual and cognitive processes related to color saliency and color vision types may manifest as distinct spatiotemporal patterns in neural activity during the oddball task. To comprehensively explore these patterns, we employed an exploratory statistical analysis technique known as cluster-based permutation analysis. This approach is nonparametric and data-driven, allowing us to assess the differences in neural activity across multiple time points and channels without predefined assumptions regarding specific time or spatial locations, while maintaining nominal Type I error rates (Maris and Oostenveld, 2007; Sassenhagen and Draschkow, 2019).

The cluster-based permutation test evaluates the null hypothesis that different conditions (e.g., different stimulus conditions or color vision types) are sampled from the same distribution; therefore, they are inter-exchangeable. If the observed effect was unlikely (<2.5% in the two-tailed test) under label-shuffled data, the hypothesis was rejected, indicating that the observed effect was not by chance. Using this method, we compared i) the stimulus color conditions for each color vision type and ii) the color vision types under the same stimulus color conditions. We utilized a set of statistical functions provided by Fieldtrip (Oostenveld et al., 2011) to perform the cluster-based permutation test. Individual mean ERPs across 64 electrodes during the 600 ms following stimulus onset were used for analyzes. This time window was selected to capture both perceptual and cognitive neural activity, including the P3 component.

In our analysis, a cluster was formed when at least two continuous time points and/or neighboring electrodes exhibited significant *t*-statistics above the critical alpha level of 0.05. Cluster size was defined as the sum of *t*-statistics within that cluster, based on temporal and spatial adjacency. Since we employed two-tailed *t*-test, clusters were classified as positive or negative based on the sign of the sum of *t*-values. The significance thresholds for these clusters were determined based on a cluster distribution generated from 10,000 random partitions of the data via the Monte Carlo method. The cluster size corresponding to a probability of 0.625% (equivalent to 2.5% with Bonferroni correction for the four repeated comparisons) at either tail of the distribution was used to set the significance threshold. The significance of each cluster size was then evaluated against these thresholds.

## 3 Results

### 3.1 Flicker photometry

The luminance of the stimuli was individually adjusted to be equiluminant with a gray background using flicker photometry prior to the oddball task. The mean luminance values for deviant 1 (blue-green), deviant 2 (red), and standard stimuli (green) were 20.49 ± 0.36, 19.74 ± 0.37, and 20.11 ± 0.12 *cd*/*m*^2^ for typical trichromats; 21.27 ± 0.086, 18.96 ± 0.12, and 20.60 ± 0.095 *cd*/*m*^2^ for anomalous trichromats; and 21.43 ± 0.30, 19.04 ± 0.32, and 20.77 ± 0.58 *cd*/*m*^2^ for the deuteranope, respectively.

Statistical comparisons based on independent *t*-tests revealed significant differences between color vision types (typical trichromats and anomalous trichromats) for all three stimuli: deviant 1 (*t*(16) = -4.68, *p* = 0.0003, Cohen's *d* = 2.35), deviant 2 (*t*(16) = 4.55, *p* = 0.0003, Cohen's *d* = 2.30), and standard (*t*(16) = -2.50, *p* = 0.024, Cohen's *d* = 1.25).

### 3.2 Behavioral results

#### 3.2.1 Behavioral performance

Three RT records were identified as outliers for typical trichromats: one from deviant 1 (919 ms) and two from deviant 2 (1,588 and 1,069 ms). All three records were from the same participant. Similarly, three RT records were identified as outliers from anomalous trichromats: once from deviant 1 (986 ms) and twice from deviant 2 (1,051 and 981 ms). These records were also from the same participant and were excluded from the behavioral analyzes.

Hit rates, which reflect the rates of successful responses to deviants, were high across all color vision types. The group mean hit rates were 99.71 and 99.42% for typical trichromats, 100 and 99.50% for anomalous trichromats, and 100% for one deuteranope participant for deviants 1 and 2, respectively. The group mean false alarm rates, which reflect the likelihood of responses to the non-target standard stimulus, were low for all color vision types: 0.1% for typical trichromats, 0.05% for anomalous trichromats, and 0.17% for deuteranopes. Due to few recorded false trials, it was impossible to identify which deviant was more likely to be falsely detected.

**Figure 3:**
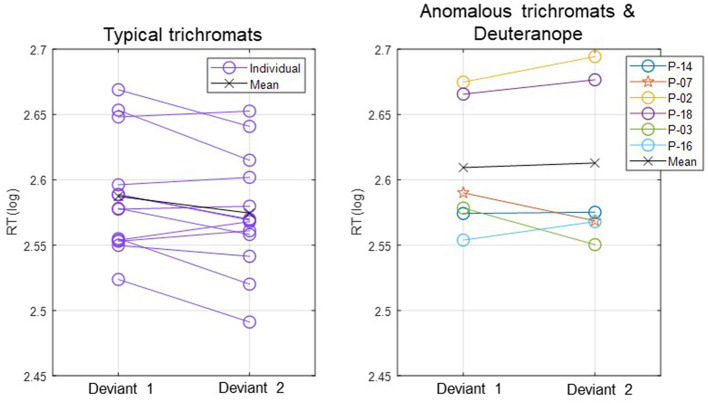
Individual mean RTs for each participant and group mean RTs for typical trichromats and anomalous trichromats for deviants 1 (blue-green) and 2 (red). For anomalous trichromats, the colors of the symbols correspond to the participants outlined in Table 1, in which participants are listed in descending order according to their red-green thresholds, as are listed in the legend of the right panel. Stars, plotted with anomalous trichromats, indicate individual mean RTs for the deuteranope while circles indicate those for typical and anomalous trichromats.

In Figure 3, log-transformed individual mean RTs are presented in two separate plots: one for typical trichromats and the other for the anomalous trichromats and deuteranope.

The participant with deuteranopia exhibited significantly faster RTs to deviant 2 (red) (2.57 ± 0.06) than deviant 1 (blue-green) (2.59 ± 0.06), indicating a faster response to the deviant that was expected to have lower saliency (*t*(158) = 2.18, *p* = 0.03, Cohen's *d* = 0.34).

#### 3.2.2 Comparative analysis with LME models

The influence of different chromatic sensitivity specifications was evaluated using a likelihood ratio test, along with AIC, BIC, and Log-Likelihood values of the LME models. We first compared the simple model, which included only stimulus condition as a fixed effect, against two more complex models: the color vision type model (which adds color vision type as a fixed effect) and the red-green threshold model (which adds the red-green threshold as a fixed effect). Both comparisons showed significant improvements in fit over the simple model, indicating that chromatic sensitivity is a crucial predictor of behavioral performance. Specifically, the color vision type model showed a significant improvement over the simple model (*p* = 0.007), while the red-green threshold model demonstrated a slightly better fit than the color vision type model (*p* = 0.006), as shown in Table 2.

**Table 2:** Results of the likelihood ratio tests comparing the simple LME model (Model 1) on RTs with two more complex models: Model 2, which includes categorical color vision type, and Model 3, which includes continuous red-green threshold as fixed effects.

**1. Simple:**	Trial-based RTs ~ Condition + (1 ∣ Participants)						
2. Color vision:	Trial-based RTs ~ ColorVision * Condition + (1 ∣ Participants)						
3. Red-green threshold:	Trial-based RTs ~ RGthreshold * Condition + (1 ∣ Participants)						
**Model**	**DF**	**AIC**	**BIC**	**Log-Likelihood**	**LRStat**	**deltaDF**	***p*** **-value**
Model 1	4	−7,212.238	−7,188.412	3,610.119			
Model 1 vs. 2	6	−7,218.213	−7,182.474	3,615.107	9.976	2	0.007^*^
Model 1 vs. 3	6	−7,218.415	−7,182.676	3,615.207	10.177	2	0.006^*^

Single asterisk indicates *p* < 0.05.

This slight improvement in fit was also reflected in the model fit indices: the red-green threshold had an AIC of –7,218.415, BIC of –7,182.676, and Log-Likelihood of 3,615.207, slightly better than the color vision type model, which had an AIC of –7,218.213, BIC of –7,182.474, and Log-Likelihood of 3,615.107 (see also Table 2). Although the two models presented similar figures, the red-green threshold model provided a more precise prediction of behavioral performance than the color vision type model.

In the red-green threshold model, a significant interaction was found between the red-green threshold and stimulus condition (*p* = 0.008), along with a significant main effect for stimulus condition (*p* = 0.001; Table 3). The interaction suggests that as the red-green threshold increased (indicating lower chromatic sensitivity), the difference in RTs between deviant 1 and deviant 2 decreased. The main effect estimate of –0.008 indicates that when the red-green threshold is at its average level (zero after *z*-scoring), RTs were generally shorter for deviant 2 compared to deviant 1.

**Table 3:** Summary of trial-based RTs analyzed using LME model with red-green threshold added as a fixed effect.

**LME model**	Trial-based RTs ~ RGthreshold * Condition + (1 ∣ Participants)						
**Fixed effect**	**Estimate**	**SE**	**tStat**	**DF**	***p*** **-value**	**95 % CI Lower**	**95 % CI Upper**
Red-green threshold	0.016	0.010	1.501	2,850	0.133	−0.005	0.036
Stimulus condition	−0.008	0.003	−3.276	2,850	0.001^*^	−0.013	−0.003
Red-green threshold: Stimulus condition	0.007	0.003	2.665	2,850	0.008^*^	0.002	0.012

The model with red-green sensitivity demonstrated better fit to the data compared to the model with color vision type. Significant interaction was found between color vision type and the stimulus condition. Single asterisk indicates *p* < xs0.05.

To confirm the effectiveness of the interaction between the red-green threshold and stimulus condition, a likelihood ratio test was conducted comparing a model with interaction term to a model without interaction term. The test indicated a significant improvement in model fit for the interaction model (*p* = 0.008), as evidenced by lower AIC (interaction model: –7,218.415, without interaction model: –7,213.323) and higher Log-Likelihood (interaction model: 3,615.208, without interaction model: 3,611.661) values.

### 3.3 Electrophysiological results

#### 3.3.1 ERPs and LME model analysis

**Figure 4:**
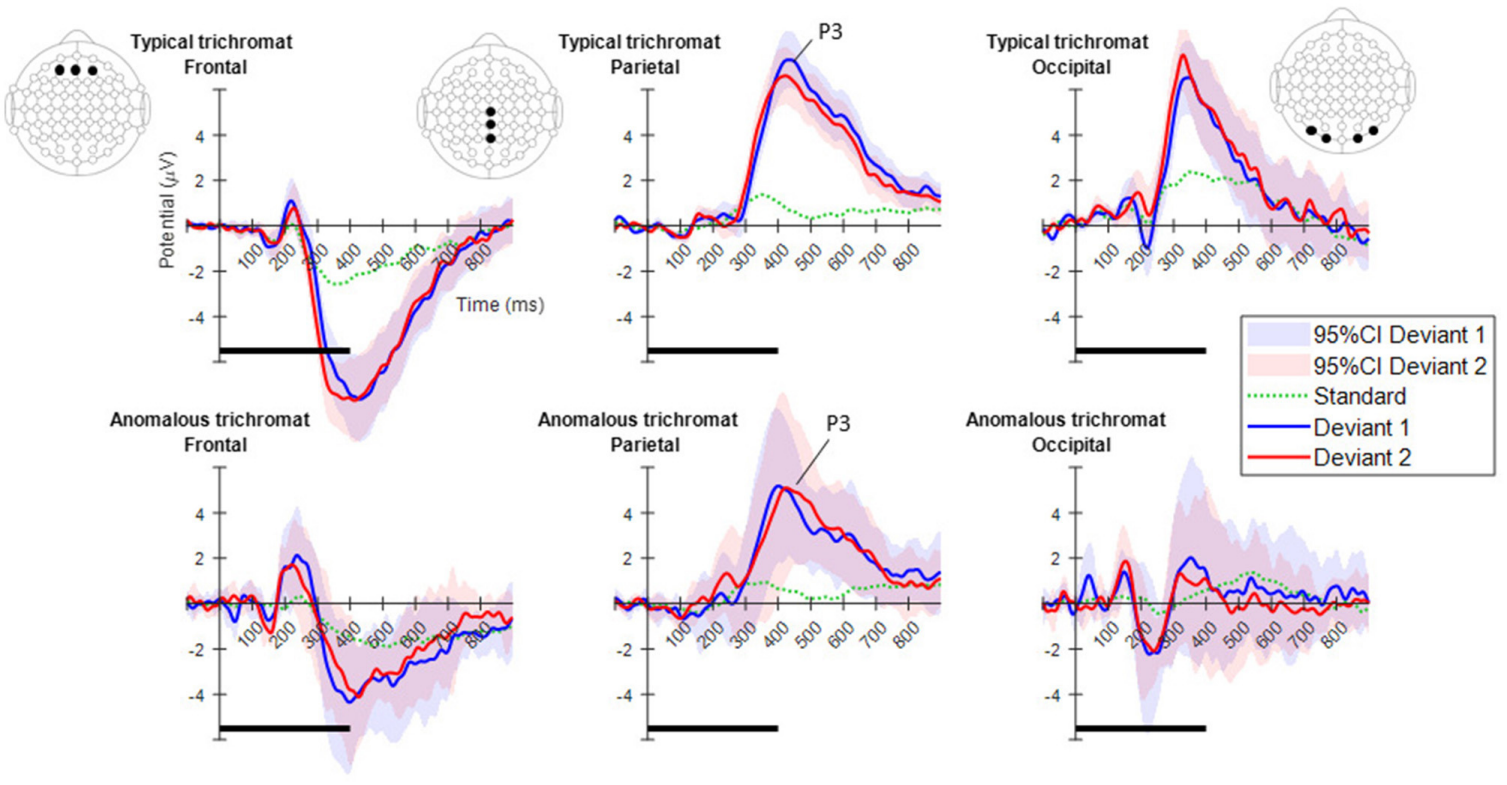
Mean ERPs for each color vision group in the three scalp regions. From left to right, ERPs are shown for the frontal, parietal, and occipital regions. The electrode positions included in each region are highlighted with black dots in the electrode layout. The plots in the top row indicate typical trichromats, while those in the bottom row indicate anomalous trichromats. In each plot, blue and red lines represent the mean ERPs across participants for deviants 1 (blue-green) and 2 (red), while shaded areas in the same color indicate 95% confidence intervals (CIs) for each deviant. A dotted green line indicates the mean potential for standard stimulus. Horizontal black lines at the bottom of each plot indicate the duration of stimulus presentation.

**Figure 5:**
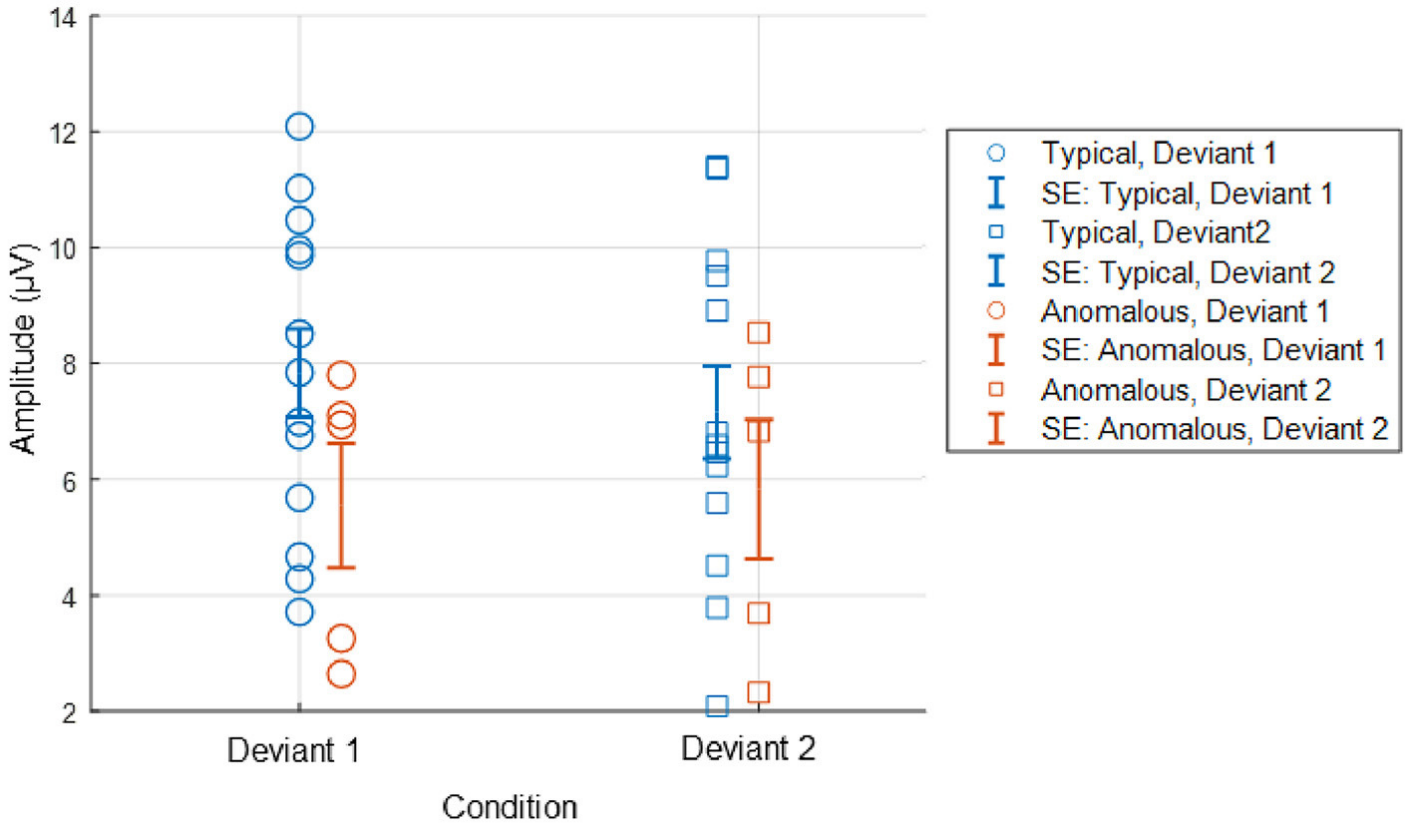
Individual P3 amplitude across stimulus conditions and color vision types. P3 amplitude was computed as the maximum positive activity during the 301–500 ms window, based on averaged centroparietal electrodes (Cz, CPz, and Pz). Deviant 1 and Deviant 2 correspond stimulus conditions of blue-green and red, respectively. Error bars in the figure represent standard error (SE).

Figure 4, displays the ERPs for each color vision group across three distinct scalp regions. The central column shows the ERPs in the parietal region, averaged across Cz, CPz, and Pz. The P3 component was clearly observed in both deviant conditions, while the response to the standard condition was suppressed in both color vision groups. The 95% confidence intervals for anomalous trichromats were generally wider than those for typical trichromats, suggesting greater variability in their responses. The comparative analysis of LME models revealed no significant improvement with the addition of chromatic sensitivity, whether specified categorically or continuously, as shown in Table 4 (simple model vs. color vision model: *p* = 0.067; simple model vs. red-green threshold model: *p* = 0.081). Despite the lack of statistical significance, the color vision type model showed the best fit among the three models, based on AIC, BIC, and Log-Likelihood values. The color vision type model did not reveal any significant main effects or interactions, indicating limited impact on P3 amplitude (Table 5). Individual mean P3 amplitude across stimulus conditions and color vision types are shown in Figure 5.

**Table 4:** Results of the likelihood ratio tests comparing the simple LME model (Model 1) on P3 amplitude with two more complex models: Model 2, which includes categorical color vision type, and Model 3, which includes continuous red-green threshold as fixed effects.

**1. Simple:**	P3 ~ Condition + (1 ∣ Participants)						
2. Color vision:	P3 ~ ColorVision * Condition + (1 ∣ Participants)						
3. Red-green threshold:	P3 ~ RGthreshold * Condition + (1 ∣ Participants)						
**Model**	**DF**	**AIC**	**BIC**	**Log- likelihood**	**LRStat**	**deltaDF**	***p*** **-value**
Model 1	4	145.540	151.874	−68.77			
Model 1 vs. 2	6	144.125	153.627	−66.063	5.415	2	0.067
Model 1 vs. 3	6	144.510	154.012	−66.255	5.030	2	0.081

**Table 5:** Summary of LME model on P3 amplitude with color vision type added as a fixed effect.

**LME model:**	P3 ~ ColorVision * Condition + (1 ∣ Participants)						
**Fixed effect**	**Estimate**	**SE**	**tStat**	**DF**	***p*** **-value**	**95% CI Lower**	**95% CI Upper**
Color vision type	2.286	1.364	1.676	32	0.103	−0.492	5.064
Stimulus condition	0.0004	0.008	0.056	32	0.491	−0.540	1.101
Color vision type: Stimuli condition	0.023	0.018	1.283	32	0.051	−1.925	0.006

The model with color vision type demonstrated better fit to the data compared to the model with red-green threshold. Neither color vision type model or red-green threshold model showed a significant improvement over the simple model.

#### 3.3.2 Exploratory analysis of ERPs

**Figure 6:**
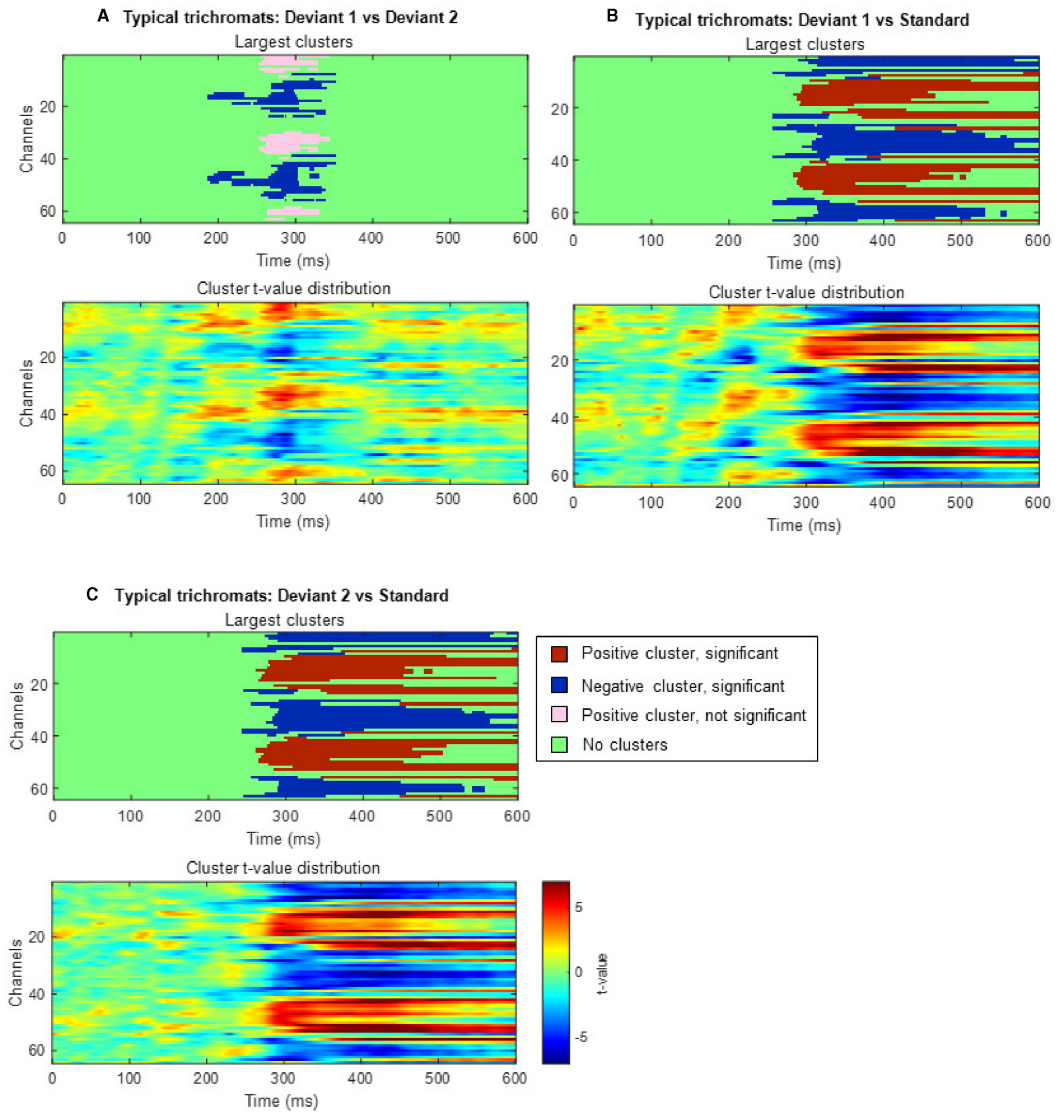
Results of the cluster-based permutation analysis for typical trichromats showing cluster distributions as a function of time and space. Each panel, **(A–C)**, consists of two sub-figures. The top sub-figure shows positive and negative clusters that showed the largest absolute cluster size in each sign. Colors of clusters indicate significance of the cluster size. The bottom sub-figure shows topographical image of *t*-value distribution. **(A)** Comparison between deviant 1 (blue-green) and deviant 2 (red) conditions. **(B)** Comparison between deviant 1 and standard (green) conditions. **(C)** Comparison between deviant 2 and standard conditions.

**Figure 7:**
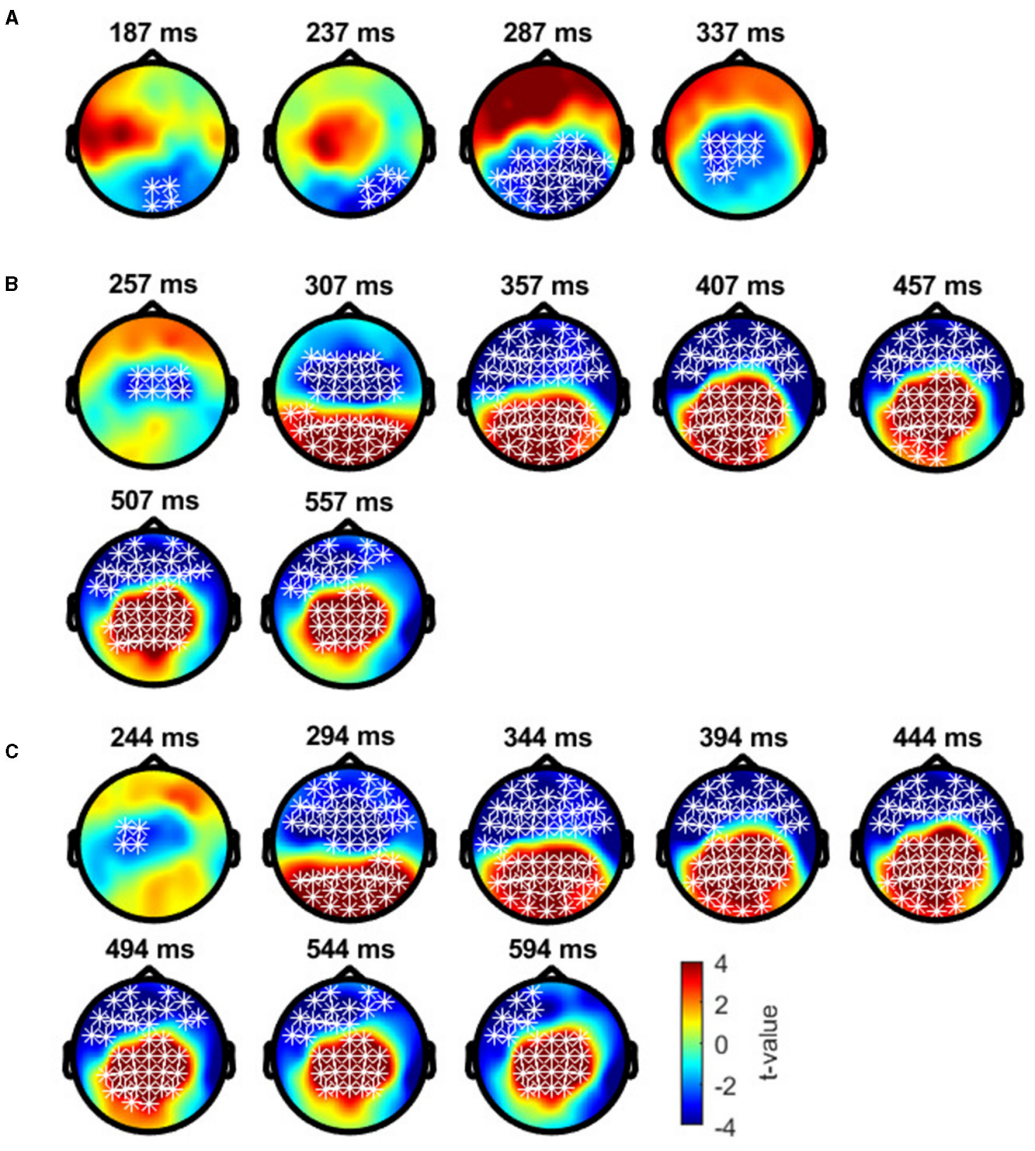
Results of the cluster-based permutation test for typical trichromats presented as scalp topography. **(A)** Comparison between deviant 1 (blue-green) and deviant 2 (red) conditions. **(B)** Comparison between deviant 1 and standard (green) conditions. **(C)** Comparison between deviant 2 and standard conditions. The distribution of *t*-values are topographically plotted, with electrodes highlighted with white asterisks to indicate locations of the clusters that exceeded the cluster size threshold with 50 ms interval.

Cluster-based permutation tests showed a significant difference between the deviant conditions in typical trichromats (Figure 6). The largest negative cluster exceeded the threshold (*p*_*adj*_ = 0.003), while the largest positive cluster was below the threshold (*p*_*adj*_ = 0.035) (Figure 6A). The electrode locations included in the negative cluster, plotted on the scalp topographies, indicated that the amplitude for deviant 2 (red) was higher than that for deviant 1 (blue-green), extended from ~200 to 350 ms post-stimulus. This cluster was predominantly distributed in the occipital region up to 240 ms, before gradually spreading and shifting toward the parietal region, with the maximal response difference occurring at ~300 ms (Figure 7A).

Comparisons of each deviant condition with the standard condition also revealed significant differences in typical trichromats (Figures 6B, C for temporal distribution and cluster size, Figures 7B, C for scalp topography). When comparing deviant 1 (blue-green) with the standard (green), the largest positive cluster, indicating a higher amplitude for deviant 1, appeared around the occipital and parietal regions (*p*_*adj*_ = 0.0004), while the largest negative cluster, indicating a lower amplitude for deviant 1 appeared around the frontal region (*p*_*adj*_ = 0.0008). Similar clusters were observed when comparing deviant 2 (red) with) the standard (positive cluster: *p*_*adj*_ = 0.0004; negative cluster: *p*_*adj*_ = 0.004). These clusters appeared ~250 ms post-stimulus and persisted until the end of the analysis window.

**Figure 8:**
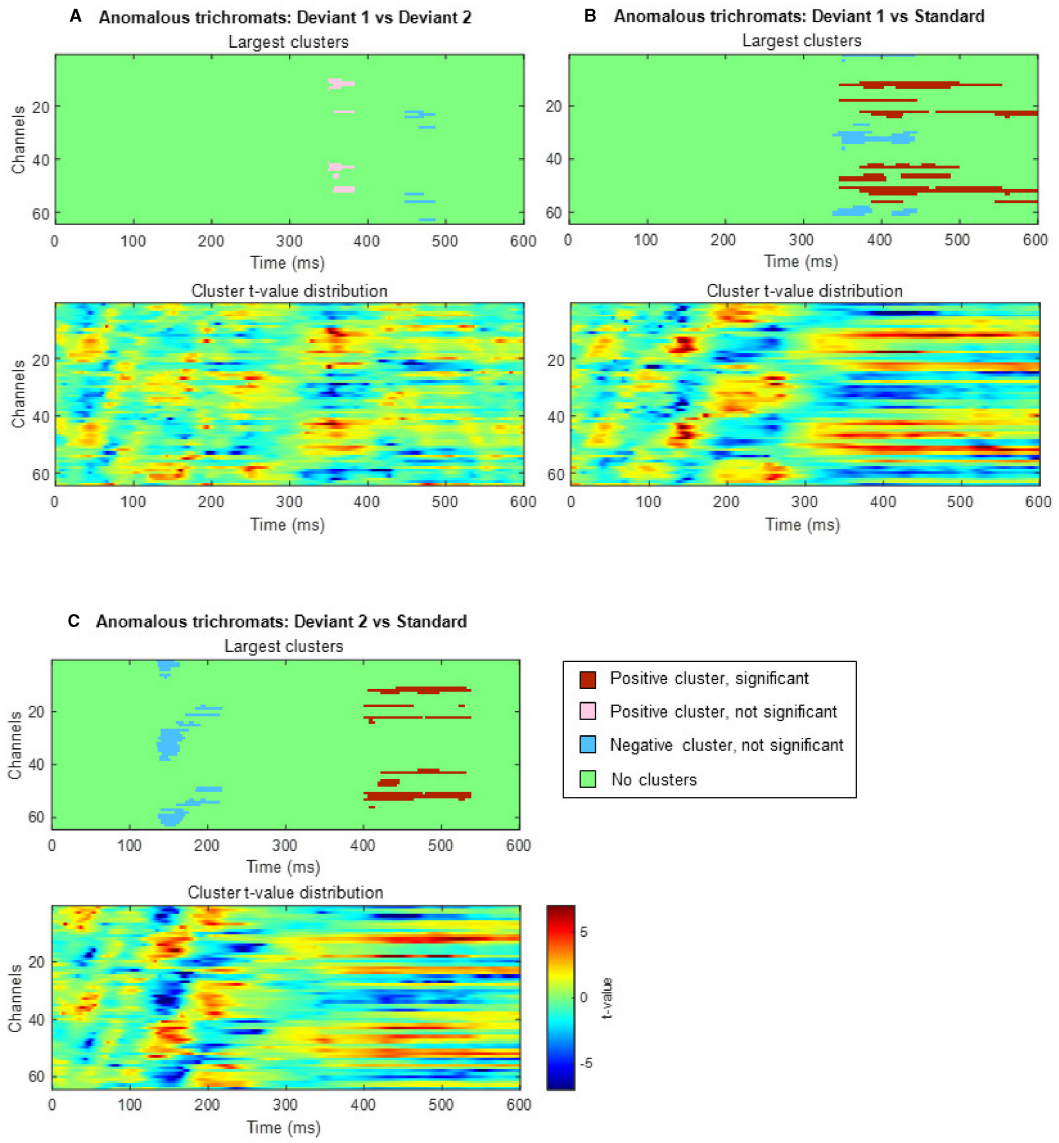
Results of the cluster-based permutation analysis for anomalous trichromats showing cluster distributions as a function of time and space. Each **(A–C)**, consists of two sub-figures. The top sub-figure shows largest positive and negative clusters that showed the largest absolute cluster size in each sign. Colors of clusters indicate significance of the cluster size. The bottom sub-figure shows topographical image of *t*-value distribution. **(A)** Comparison between deviant 1 (blue-green) and deviant 2 (red) conditions. **(B)** Comparison between deviant 1 and standard conditions. **(C)** Comparison between deviant 2 and standard conditions.

**Figure 9:**
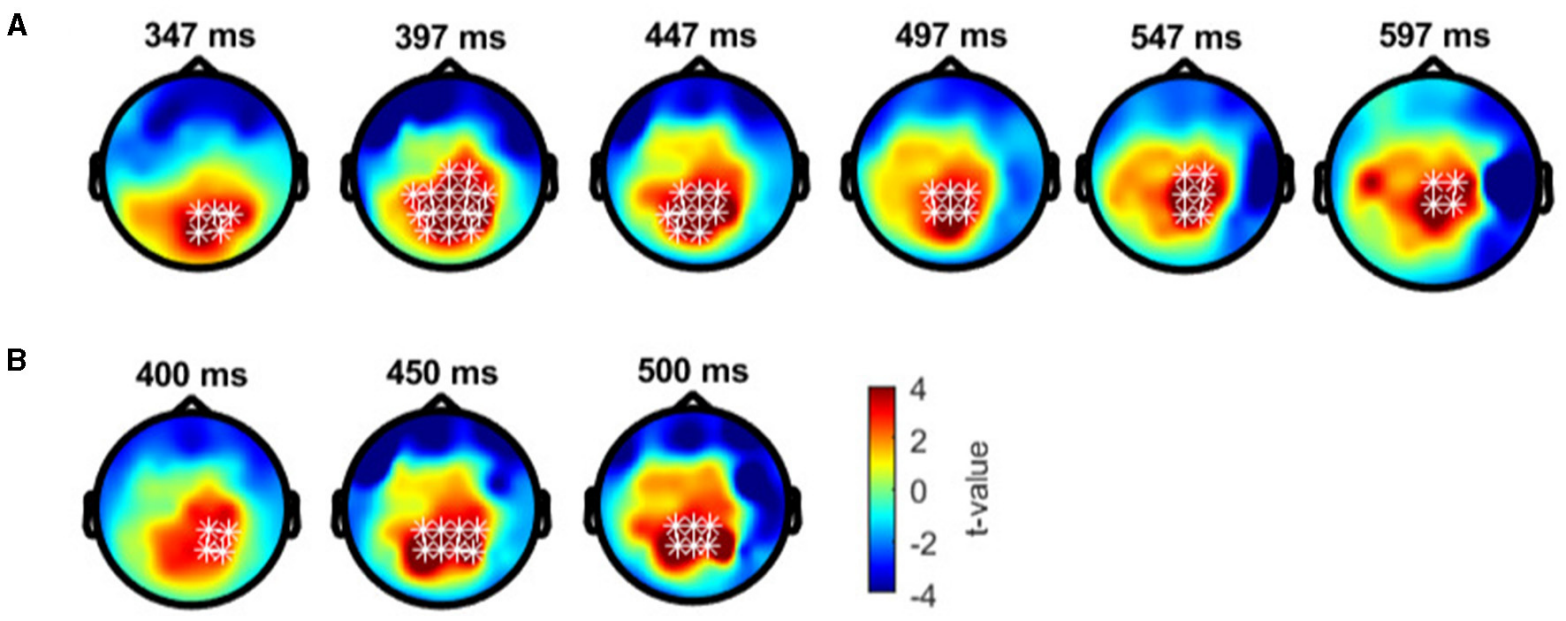
Results of the cluster-based permutation test for anomalous trichromats presented as scalp topography. **(A)** Comparison between deviant 1 (blue-green) and standard (green) conditions. **(B)** Comparison between deviant 2 (red) and standard conditions. The distribution of *t*-values are topographically plotted, with electrodes highlighted with white asterisks to indicate locations of the clusters that exceeded the cluster size threshold with 50 ms interval.

In anomalous trichromats, no significant differences were found between the deviant conditions, as no clusters exceeded the threshold (positive cluster: *p*_*adj*_ = 0.12; negative cluster: *p*_*adj*_ = 0.88; Figure 8A). However, a significant difference was observed when comparing deviant 1 (blue-green) with the standard (green; Figure 8B). A positive cluster, indicating a higher amplitude for deviant 1, appeared around the occipital to parietal regions ~350 ms post-stimulus, lasting until the end of the analysis window (positive cluster: *p*_*adj*_ = 0.0004; negative cluster: *p*_*adj*_ = 0.13; Figure 9A). Similarly, a significant difference was observed when comparing deviant 2 (red) with the standard (Figure 8C), with a positive cluster indicating a higher amplitude for deviant 2 appearing around the occipital to parietal regions ~400–500 ms post-stimulus (positive cluster: *p*_*adj*_ = 0.0004; negative cluster: *p*_*adj*_ = 0.12; Figure 9B).

**Figure 10:**
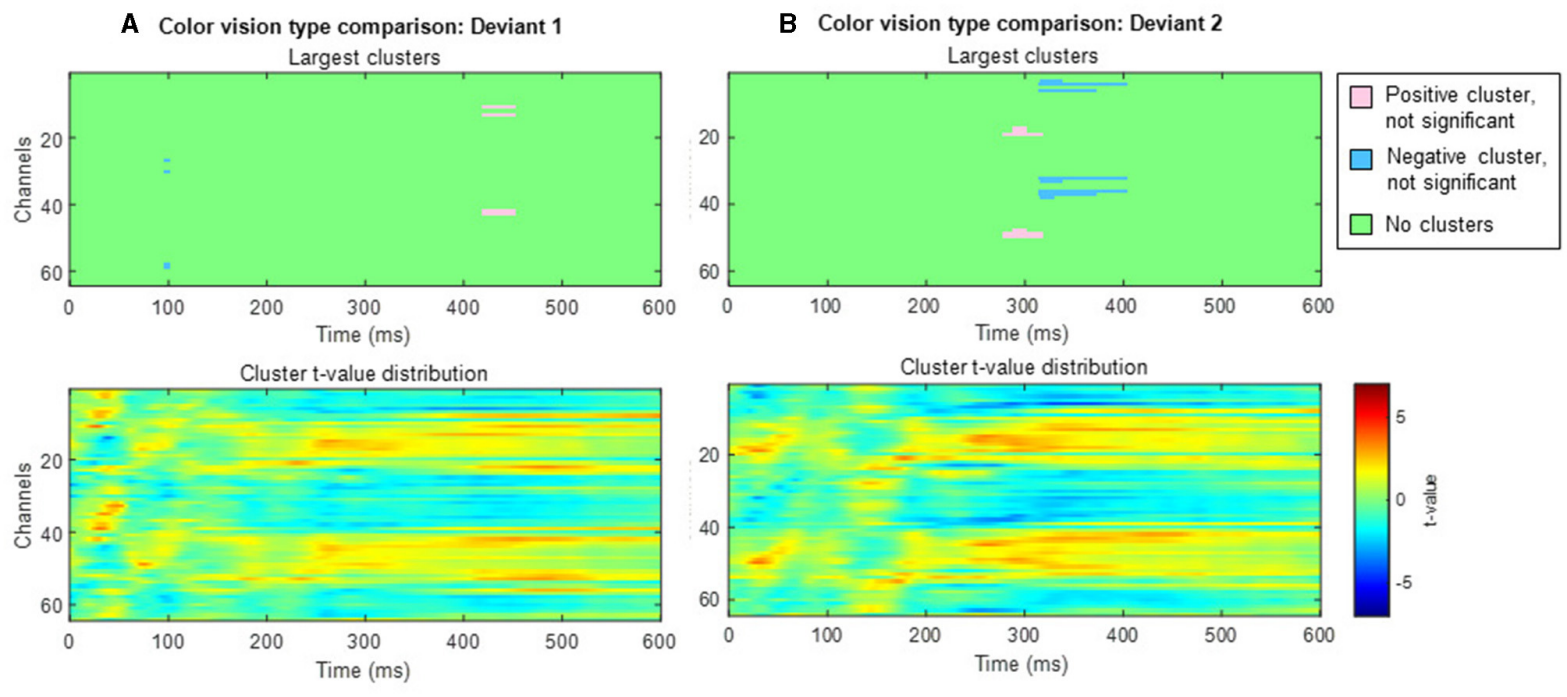
Results of the cluster-based permutation test comparing typical trichromats and anomalous trichromats. Each panel consists of two sub-figures. The top sub-figure shows largest positive and negative clusters that showed the largest absolute cluster size in each sign. Colors of clusters indicate significance of the cluster size. The bottom sub-figure shows topographical image of *t*-value distribution. **(A)** Comparison of deviant 1 (blue-green). **(B)** Comparison of deviant 2 (red).

When comparing typical and anomalous trichromats using the cluster-based permutation test, no significant differences were found for either deviant condition (deviant 1 positive cluster: *p*_*adj*_ = 1, negative cluster: *p*_*adj*_ = 1; deviant 2 positive cluster: *p*_*adj*_ = 0.14, negative cluster: *p*_*adj*_ = 0.66; Figure 10).

## 4 Discussion

This study investigated the neural responses to color saliency variations during an attention-demanding oddball task, focusing particularly on anomalous trichromats with varied red-green chromatic sensitivity. Our primary aim was to elucidate the spatiotemporal characteristics of the neural activity underlying the perceptual and cognitive processes involved in this task. Specifically, we explored the neural activities that link red-green sensitivity to attention-demanding cognitive behavior.

To facilitate consistent comparisons, we used uniform chromaticity for color stimuli across participants. Two deviant stimuli (blue-green and red) were employed to investigate how differences in chromatic sensitivity influence perceptual and cognitive processes, particularly in the context of reverse saliency conditions between typical trichromats and anomalous trichromats.

Given the need for a more nuanced analysis, LME models were employed to assess the effects of color vision type, the red-green threshold, and stimulus condition on RTs and the P3 component. The use of cluster-based permutation analyzes further explored the spatiotemporal characteristics related to red-green saliency during the perceptual and cognitive processing of colors.

The LME model analysis revealed that RTs were better described by continuous variation in chromatic sensitivity than by categorical distinctions between color vision types, as indicated by a more accurate and parsimonious model fit. This finding suggests that continuous chromatic sensitivity provides a more detailed understanding of its influence on behavioral performance, capturing aspects that categorical classification might overlook. The LME model, which included the red-green threshold, revealed a significant interaction between stimulus condition and the red-green threshold.

This interaction indicates that as red-green threshold increased—characteristic of anomalous trichromats—the differences in RTs between deviant 1 and deviant 2 were reduced. The main effect shows generally faster RTs for deviant 2, which may primarily reflect the responses of typical trichromats who perceived this condition as more salient. However, individual variability within the anomalous trichromat group suggests that the interaction effect represents an overall trend rather than a uniform response pattern among those with higher red-green thresholds. This partially confirms the effectiveness of our stimulus design. Still, the comparable RTs for both deviants and the low false alarm rate in anomalous trichromats, may indicate the presence of neural mechanisms that enhance performance beyond the constraints of red-green sensitivity. Alternatively, it could also indicate that the task was not sufficiently challenging to reveal more nuanced perceptual differences in performance.

Moreover, the relationship between RTs and chromatic sensitivity in minority color vision phenotypes was not straightforward. For instance, as shown in Figure 3, anomalous trichromat participant (P-14) with the highest red-green threshold had RTs as fast as the quicker typical trichromat participants. Additionally, a participant with deuteranopic dichromacy (P-7) exhibited faster RTs to the red stimulus, contrary to the expectations based on lower red-green sensitivity. These finding indicate that adjusting stimulus chromaticities based solely on red-green sensitivity may not yield expected outcomes, highlighting complexity of perceptual and cognitive factors in these phenotypes. This complexity aligned with previous research (Bosten, 2019), suggesting that behavioral performance of minority color vision phenotypes is influenced by a combination of perceptual and cognitive factors. For a more detailed analysis on RTs in relation to the red-green threshold, refer to the Supplementary material.

The analysis of the P3 component through LME models revealed that chromatic sensitivity had a limited impact on P3 amplitude, contrary to the prior expectations. No significant influence of stimulus condition or color vision type on P3 amplitude was found. However, the exploratory ERP analysis revealed broader spatiotemporal differences across conditions and color vision types. The cluster-based permutation test identified a cluster with higher potentiation for the more salient red deviant compared to the blue-green deviant in typical trichromats. This finding, while not fully aligning with our hypotheses, showed a temporal trend of faster neural amplification for the more salient red stimulus early in the P3 response, aligning with one of our predictions and supporting the validity of our experimental paradigm.

In contrast, the effect of saliency differences was more ambiguous in minority color vision phenotypes, evidenced by greater variability in neural responses. The cluster-based permutation analysis did not reveal clear temporal or spatial characteristics tuned to saliency differences. This ambiguity might stem from inadequate accounting for continuous variation in chromatic sensitivities. Nonetheless, during the deviant-standard comparison analysis, a cluster reflecting higher potentiation to the blue-green stimulus appeared at ~350 ms post-stimulus, while those for the red stimulus appeared ~400 ms, predominantly in the occipital region and extending to the parietal region. Although cluster-based permutation tests do not precisely pinpoint the timing and location of distinct neural activities between conditions (Sassenhagen and Draschkow, 2019), this slight timing discrepancy may relate to previously reported faster RTs to the blue-green stimulus in visual search tasks in individuals with anomalous trichromacy (Sunaga et al., 2013).

While LME models did not show a significant effect of color vision type or stimulus condition on P3 amplitude, differences between stimulus conditions were detected by the cluster-based permutation analyzes within the overlapping P3 time window. Consistent with predictions, both color vision types tended to respond faster to more salient stimuli, although higher potentiation to less salient stimuli was not observed. These results suggest a broader spatiotemporal examination of neural activity is effective in capturing attentional neural representations related to color saliency differences. Careful consideration of chromatic sensitivity variations, which may obscure the effect of saliency differences, combined with broader analyzes, can lead to a more precise understanding of cortical representation of colors in individuals with minority color vision phenotypes.

It is important to note that large individual differences in ERP waveforms were observed in both typical and anomalous trichromats (see the Supplementary material). Anatomical factors, such as skull thickness, may obscure the extent to which these differences reflect true neuronal activity (Hakim et al., 2021). Moreover, EEG signals primarily reflect pyramidal neuron activity, with minimal contribution from interneuron activity (Luck, 2014). In addition to physiological factors, attention-related neural activity is susceptible to individual variations (Kane and Engle, 2002; Curran et al., 2001), which may have been further complicated by differences in chromatic sensitivity. Furthermore, the limited sample size, particularly for minority color vision phenotypes, reduced the analytical power of this study, especially in the analysis of the P3 component, which could only be assessed after averaging, unlike RTs. These confounding factors, along with a smaller stimulus size, likely contributed to the difficulties in detecting neural representations of enhanced sensitivities in the early visual cortex, as reported in previous studies (Rabin et al., 2018; Tregillus et al., 2021). Consequently, these factors may have hindered our ability to isolate neural activity specific to saliency differences in this study.

Another limitation of this study is the uncertainty regarding the origin of the variation in red-green sensitivity—whether it arises from genetic factors or neuroplastic changes during development. Clarifying the relationship between perceptual diversity and variations in neural activity requires disentangling genetic factors, such as those related to cone sensitivities, from developmental influences, or exploring their interplay. Integrating these factors into future studies will be critical for obtaining a more comprehensive understanding of the relationship between neural, perceptual, and cognitive diversity.

## 5 Conclusion

The present study investigated the spatiotemporal dynamics of neural activity related to chromatic differences during an attention-demanding task in both anomalous and typical trichromats. In typical trichromats, distinct neural differences between color conditions were observed, with neural signals aligning with the expected saliency of the stimuli. Anomalous trichromats exhibited a similar temporal pattern, showing slightly faster neural responses to the color expected to be more salient. Behavioral reaction times were influenced by participants' red-green threshold, indicating that chromatic sensitivity was associated with behavioral variation. However, no significant differences in neural responses were found between the color vision types, despite sensitivity differences. These findings underscore the complex relationship between red-green sensitivity, neural responses, and behavior. Further study with larger sample sizes is needed to more comprehensively characterize neural activity related to perceptual, cognitive, and behavioral processing across color vision types.

## Data Availability

The original contributions presented in the study are included in the article/Supplementary material, further inquiries can be directed to the corresponding author.

## References

[B1] Aho K. Derryberry D. Peterson T. (2014). Model selection for ecologists: the worldviews of AIC and BIC. Ecology 95, 631–636. 10.1890/13-1452.1 24804445

[B2] Alho K. Woods D. L. Algazi A. Näätänen R. (1992). Intermodal selective attention. ii. effects of attentional load on processing of auditory and visual stimuli in central space. Electroencephal. Clin. Neurophysiol. 82, 356–368. 10.1016/0013-4694(92)90005-3 1374704

[B3] Anstis S. Cavanagh P. (1983). A Minimum Motion Technique for Judging Equiluminance. York University, Toronto, ON.

[B4] Asenjo A. B. Rim J. Oprian D. D. (1994). Molecular determinants of human red/green color discrimination. Neuron 12, 1131–1138. 8185948 10.1016/0896-6273(94)90320-4

[B5] Barbur J. L. Rodriguez-Carmona M. (2017). Colour vision requirements in visually demanding occupations. Br. Med. Bullet. 122, 51–77. 10.1093/bmb/ldx007 28334313

[B6] Barbur J. L. Rodriguez-Carmona M. Evans B. E. (2021). Color vision assessment-3. an efficient, two-step, color assessment protocol. Color Res. Appl. 46, 33–45. 10.1002/col.22599

[B7] Berlin B. Kay P. (1991). Basic color terms: Their Universality and Evolution. University of California Press.

[B8] Birch J. (2012). Worldwide prevalence of red-green color deficiency. JOSA A 29, 313–320. 10.1364/JOSAA.29.000313 22472762

[B9] Boehm A. E. Bosten J. MacLeod D. I. (2021). Color discrimination in anomalous trichromacy: experiment and theory. Vis. Res. 188, 85–95. 10.1016/j.visres.2021.05.011 34293614

[B10] Bosten J. (2019). The known unknowns of anomalous trichromacy. Curr. Opin. Behav. Sci. 30, 228–237. 10.1016/j.cobeha.2019.10.015

[B11] Brainard D. H. Vision S. (1997). The psychophysics toolbox. Spat. Vis. 10, 433–436. 9176952

[B12] Broackes J. (2010). Unilateral colour vision defects and the dimensions of dichromat experience. Ophthal. Physiol. Opt. 30, 672–684. 10.1111/j.1475-1313.2010.00774.x 20883354

[B13] Buscemi S. Plaia A. (2020). Model selection in linear mixed-effect models. Adv. Statist. Anal. 104, 529–575. 10.1007/s10182-019-00359-z

[B14] Conway B. R. Malik-Moraleda S. Gibson E. (2023). Color appearance and the end of hering's opponent-colors theory. Trends Cogn. Sci. 6:3. 10.1016/j.tics.2023.06.003 37394292 PMC10527909

[B15] Curran T. Hills A. Patterson M. B. Strauss M. E. (2001). Effects of aging on visuospatial attention: an ERP study. Neuropsychologia 39, 288–301. 10.1016/S0028-3932(00)00112-3 11163607

[B16] Dartnall H. J. Bowmaker J. K. Mollon J. D. (1983). Human visual pigments: microspectrophotometric results from the eyes of seven persons. Proc. Royal Soc. Lond. Ser. B Biol. Sci. 220, 115–130. 6140680 10.1098/rspb.1983.0091

[B17] Deeb S. (2005). The molecular basis of variation in human color vision: variation in human color vision. Clin. Genet. 65, 369–377. 10.1111/j.1399-0004.2004.00343.x 15811001

[B18] Delorme A. (2023). EEG is better left alone. Sci. Rep. 13:2372. 10.1038/s41598-023-27528-0 36759667 PMC9911389

[B19] Delorme A. Makeig S. (2004). EEGlab: an open source toolbox for analysis of single-trial EEG dynamics including independent component analysis. J. Neurosci. Methods 134, 9–21. 10.1016/j.jneumeth.2003.10.009 15102499

[B20] Elliot A. J. Maier M. A. (2007). Color and psychological functioning. Curr. Direct. Psychol. Sci. 16, 250–254. 10.1111/j.1467-8721.2007.00514.x

[B21] Gorgolewski K. J. Auer T. Calhoun V. D. Craddock R. C. Das S. Duff E. P.. (2016). The brain imaging data structure, a format for organizing and describing outputs of neuroimaging experiments. Sci. Data 3, 1–9. 10.1038/sdata.2016.44 27326542 PMC4978148

[B22] Grasso D. J. Moser J. S. Dozier M. Simons R. (2009). ERP correlates of attention allocation in mothers processing faces of their children. Biol. Psychol. 81, 95–102. 10.1016/j.biopsycho.2009.03.001 19428973 PMC3422636

[B23] Gray H. M. Ambady N. Lowenthal W. T. Deldin P. (2004). P300 as an index of attention to self-relevant stimuli. J. Exp. Soc. Psychol. 40, 216–224. 10.1016/S0022-1031(03)00092-1

[B24] Hakim N. Awh E. Vogel E. K. Rosenberg M. D. (2021). Inter-electrode correlations measured with EEG predict individual differences in cognitive ability. Curr. Biol. 31, 4998–5008. 10.1016/j.cub.2021.09.036 34637747 PMC8612967

[B25] Hiramatsu C. Takashima T. Sakaguchi H. Chen X. Tajima S. Seno T.. (2023). Influence of colour vision on attention to, and impression of, complex aesthetic images. Proc. Royal Soc. B 290:20231332. 10.1098/rspb.2023.1332 37700648 PMC10498032

[B26] Isreal J. B. Wickens C. D. Donchin E. (1980). The dynamics of p300 during dual-task performance. Progr. Brain Res. 54, 416–421. 10.1016/S0079-6123(08)61653-2 7220944

[B27] Jordan G. Deeb S. S. Bosten J. M. Mollon J. D. (2010). The dimensionality of color vision in carriers of anomalous trichromacy. J. Vis. 10:12. 10.1167/10.8.12 20884587

[B28] Kaiser P. K. Comerford J. P. (1975). Flicker photometry of equally bright lights. Vis. Res. 15, 1399–1402. 1210025 10.1016/0042-6989(75)90197-2

[B29] Kane M. J. Engle R. W. (2002). The role of prefrontal cortex in working-memory capacity, executive attention, and general fluid intelligence: an individual-differences perspective. Psychon. Bullet. Rev. 9, 637–671. 10.3758/BF03196323 12613671

[B30] Kay P. Regier T. (2003). Resolving the question of color naming universals. Proc. Natl. Acad. Sci. U. S. A. 100, 9085–9089. 10.1073/pnas.1532837100 12855768 PMC166442

[B31] Keil A. Debener S. Gratton G. Junghöfer M. Kappenman E. S. Luck S. J.. (2014). Committee report: publication guidelines and recommendations for studies using electroencephalography and magnetoencephalography. Psychophysiology 51, 1–21. 10.1111/psyp.12147 24147581

[B32] Kleiner M. Brainard D. Pelli D. Ingling A. Murray R. Broussard C. (2007). Whats new in psychtoolbox-3? *Perception* 36:2007.

[B33] Kramer A. Schneider W. Fisk A. Donchin E. (1986). The effects of practice and task structure on components of the event-related brain potential. Psychophysiology 23, 33–47. 3945706 10.1111/j.1469-8986.1986.tb00590.x

[B34] Luck S. J. (2014). An Introduction to the Event-Related Potential Technique. Cambridge, MA: MIT Press.

[B35] Lundberg J. (2007). Lifting the crown—citation *z*-score. *J. Informetr*. 1, 145–154. 10.1016/j.joi.2006.09.007

[B36] Maris E. Oostenveld R. (2007). Nonparametric statistical testing of EEG-and MEG-data. J. Neurosci. Methods 164, 177–190. 10.1016/j.jneumeth.2007.03.024 17517438

[B37] Merbs S. L. Nathans J. (1992). Absorption spectra of the hybrid pigments responsible for anomalous color vision. Science 258, 464–466. 1411542 10.1126/science.1411542

[B38] Montag E. D. (1994). Surface color naming in dichromats. Vis. Res. 34, 2137–2151. 7941411 10.1016/0042-6989(94)90323-9

[B39] Montag E. D. Boynton R. M. (1987). Rod influence in dichromatic surface color perception. Vis. Res. 27, 2153–2162. 3502300 10.1016/0042-6989(87)90129-5

[B40] Muratbekova M. Shamoi P. (2024). Color-emotion associations in art: fuzzy approach. IEEE Access 2024:3375361. 10.1109/ACCESS.2024.3375361

[B41] Murray M. M. Brunet D. Michel C. M. (2008). Topographic ERP analyses: a step-by-step tutorial review. Brain Topogr. 20, 249–264. 10.1007/s10548-008-0054-5 18347966

[B42] Näätänen R. (1988). Implications of ERP data for psychological theories of attention. Biol. Psychol. 26, 117–163. 3061477 10.1016/0301-0511(88)90017-8

[B43] Nagy A. L. Boynton R. M. (1979). Large-field color naming of dichromats with rods bleached. JOSA 69, 1259–1265. 316449 10.1364/josa.69.001259

[B44] Nathans J. Piantanida T. P. Eddy R. L. Shows T. B. Hogness D. S. (1986). Molecular genetics of inherited variation in human color vision. Science 232, 203–210. 3485310 10.1126/science.3485310

[B45] Neitz J. Neitz M. (2011). The genetics of normal and defective color vision. Vis. Res. 51, 633–651. 10.1016/j.visres.2010.12.002 21167193 PMC3075382

[B46] Neitz J. Neitz M. He J. Shevell S. (1999). Trichromatic color vision with only two spectrally distinct photopigments. Nat. Neurosci. 2, 884–888. 10491608 10.1038/13185

[B47] Okajima K. (2011). “Color vision deficiency,” in Handbook of Color Science, 3rd Edn (in Japanese), ed. The Color Science Association of Japan (Tokyo: The University of Tokyo Press), 380–403.

[B48] Oostenveld R. Fries P. Maris E. Schoffelen J.-M. (2011). Fieldtrip: open source software for advanced analysis of MEG, EEG, and invasive electrophysiological data. Comput. Intell. Neurosci. 2011, 1–9. 10.1155/2011/156869 21253357 PMC3021840

[B49] Pelli D. G. Vision S. (1997). The videotoolbox software for visual psychophysics: transforming numbers into movies. Spat. Vis. 10, 437–442. 9176953

[B50] Pernet C. R. Appelhoff S. Gorgolewski K. J. Flandin G. Phillips C. Delorme A.. (2019). EEG-bids, an extension to the brain imaging data structure for electroencephalography. Sci. Data 6:103. 10.1038/s41597-019-0104-8 31239435 PMC6592877

[B51] Picton T. W. (1992). The p300 wave of the human event-related potential. J. Clin. Neurophysiol. 9, 456–456. 1464675 10.1097/00004691-199210000-00002

[B52] Pinheiro J. C. Bates D. M. (1995). Approximations to the log-likelihood function in the nonlinear mixed-effects model. J. Comput. Graph. Statist. 4, 12–35.19163823

[B53] Pointer M. (1981). A comparison of the cie 1976 colour spaces. Color Res. Appl. 6, 108–118.

[B54] Pokorny J. Smith V. C. (1977). Evaluation of single-pigment shift model of anomalous trichromacy. JOSA 67, 1196–1209. 409816 10.1364/josa.67.001196

[B55] Polich J. (1987). Task difficulty, probability, and inter-stimulus interval as determinants of p300 from auditory stimuli. Electroencephal. Clin. Neurophysiol. 68, 311–320. 2439311 10.1016/0168-5597(87)90052-9

[B56] Pridmore R. W. (2014). Orthogonal relations and color constancy in dichromatic colorblindness. PLoS ONE 9:e107035. 10.1371/journal.pone.0107035 25211128 PMC4161355

[B57] Rabin J. Kryder A. Lam D. (2018). Binocular facilitation of cone-specific visual evoked potentials in colour deficiency. Clin. Exp. Optomet. 101, 69–72. 10.1111/cxo.12567 28636141

[B58] Sassenhagen J. Draschkow D. (2019). Cluster-based permutation tests of MEG/EEG data do not establish significance of effect latency or location. Psychophysiology 56:e13335. 10.1111/psyp.13335 30657176

[B59] Scheibner H. M. Boynton R. M. (1968). Residual red-green discrimination in dichromats. JOSA 58, 1151–1158. 10.1364/josa.58.001151 5668366

[B60] Schielzeth H. (2010). Simple means to improve the interpretability of regression coefficients. Methods Ecol. Evol. 1, 103–113. 10.1111/j.2041-210X.2010.00012.x18450400

[B61] Smith V. C. Pokorny J. (1977). Large-field trichromacy in protanopes and deuteranopes. JOSA 67, 213–220. 10.1364/josa.67.000213 300100

[B62] Stockman A. Sharpe L. T. (2000). The spectral sensitivities of the middle-and long-wavelength-sensitive cones derived from measurements in observers of known genotype. Vis. Res. 40, 1711–1737. 10.1016/S0042-6989(00)00021-3 10814758

[B63] Sunaga S. Ogura T. Seno T. (2013). Evaluation of a dichromatic color-appearance simulation by a visual search task. Opt. Rev. 20, 83–93. 10.1007/s10043-013-0013-6

[B64] Sutton S. Braren M. Zubin J. John E. (1965). Evoked-potential correlates of stimulus uncertainty. Science 150, 1187–1188. 5852977 10.1126/science.150.3700.1187

[B65] Takahashi N. Sawayama M. Chen X. Motomura Y. Takeichi H. Miyauchi S.. (2023). Temporal and spatial analysis of event-related potentials in response to color saliency differences among various color vision types. bioRxiv. 10.1101/2023.09.12.557493 PMC11479979 39416684

[B66] Thomas P. Formankiewicz M. Mollon J. (2011). The effect of photopigment optical density on the color vision of the anomalous trichromat. Vis. Res. 51, 2224–2233. 10.1016/j.visres.2011.08.016 21893078

[B67] Tregillus K. E. Isherwood Z. J. Vanston J. E. Engel S. A. MacLeod D. I. Kuriki I.. (2021). Color compensation in anomalous trichromats assessed with fMRI. Curr. Biol. 31, 936–942. 10.1016/j.cub.2020.11.039 33326771 PMC7946702

[B68] Vuong Q. H. (1989). Likelihood ratio tests for model selection and non-nested hypotheses. Econometrica 1989, 307–333.

[B69] Wagner G. Boynton R. M. (1972). Comparison of four methods of heterochromatic photometry. JOSA 62, 1508–1515. 10.1364/josa.62.001508 4643012

[B70] Werner J. S. Wooten B. R. (1979). Opponent chromatic mechanisms: relation to photopigments and hue naming. JOSA 69, 422–434. 458509 10.1364/josa.69.000422

[B71] Witzel C. Gegenfurtner K. R. (2015). Categorical facilitation with equally discriminable colors. J. Vis. 15:22. 10.1167/15.8.22 26129860

[B72] Zhang W. Kappenman E. S. (2024). Maximizing signal-to-noise ratio and statistical power in ERP measurement: single sites versus multi-site average clusters. Psychophysiology 61:e14440. 10.1111/psyp.14440 37973199

